# Expression of m6A Regulators Correlated With Immune Microenvironment Predicts Therapeutic Efficacy and Prognosis in Gliomas

**DOI:** 10.3389/fcell.2020.594112

**Published:** 2020-11-10

**Authors:** Shengchao Xu, Lu Tang, Gan Dai, Chengke Luo, Zhixiong Liu

**Affiliations:** ^1^Department of Neurosurgery, Xiangya Hospital of Central South University, Changsha, China; ^2^Department of Thoracic Surgery, Xiangya Hospital of Central South University, Changsha, China; ^3^Department of Microbiology, Xiangya School of Medicine, Central South University, Changsha, China

**Keywords:** glioma, brain tumor, N6-methyladenosine methylation, immune microenvironment, immune infiltration, chemoradiotherapy

## Abstract

**Background:**

N6-methyladenosine (m6A) RNA methylation and tumor immune microenvironment played crucial roles in cancer development. However, their association in gliomas remains to be fully elucidated.

**Methods:**

A total of 2144 glioma patients from CGGA, TCGA, and Rembrandt databases were extracted in our study, in which 325 were set as the training cohort and 1819 were defined as the validation cohort. Survival differences evaluated by Kaplan–Meier analysis between groups. Patients were clustered into subgroups by consensus clustering. ESTIMATE algorithm was applied to calculate immune and stroma scores. The infiltration of immune cells was characterized by TIMER algorithm. The risk signature was constructed by multivariate Cox regression analysis.

**Results:**

Nineteen m6A regulators were highly expressed in glioma tissues. The expression of m6A regulators was associated with prognoses, grade, isocitrate dehydrogenase (IDH) status, and 1p19q status of gliomas. Two subgroups were identified by consensus clustering, in which cluster 1 was associated with favorable prognosis, high stroma and immune scores, and high immune infiltration. When the patients were divided into high risk and low risk groups based on their risk scores, we found that patients in the high risk group had poor prognoses. Besides, patients in the high risk group had a higher stroma and immune scores, and higher abundance of immune infiltration. These results were further verified in the validation cohort, which contained three independent datasets. Moreover, patients in the low risk group enjoyed better prognoses without chemoradiotherapy or single chemotherapy.

**Conclusion:**

Our study revealed that m6A regulators could predict the prognosis and therapeutic efficacy, and were also associated with the immune microenvironment in gliomas.

## Introduction

Gliomas are the most common primary malignancies in the central nervous system. The morbidity of gliomas is approximately 7 cases per 100,000 people, which accounts for the majority of primary brain tumors (Ostrom et al., 2019). Based on the World Health Organization (WHO) classification, gliomas are classified into four grades, in which grade 1 and grade 2 gliomas are defined as low-grade glioma whereas grade 3 and grade 4 gliomas are termed as high-grade glioma (Louis et al., 2016). Typically, patients with a higher grade glioma suffer from a worse prognosis. The 10-year survival rate of patients with low-grade glioma is 47% with a median survival time of 11.6 years (Ohgaki and Kleihues, 2005; Smoll et al., 2012). Although the low-grade glioma is relatively more optimistic compared to high-grade glioma, almost 70% of low-grade glioma will progress to high-grade one in a few years (Maher et al., 2001). The median survival time of patients with grade 3 glioma is approximately 3 years and that of patients with grade 4 glioma is about 13 months (Bleeker et al., 2012). Since patients with gliomas have such a poor prognosis, there is a clear urgent to find novel biomarkers to predict the prognosis. Previous studies have found that gliomas with isocitrate dehydrogenase (IDH) mutation and 1p19q codeletion indicate a relatively favorable survival (Eckel-Passow et al., 2015). Besides, O-6-methylguanine-DNA methyltransferase (MGMT) promoter methylation has been found to increase the chemosensitivity of temozolomide treatment, and it is a strong prognostic biomarker in patients with glioblastoma (Wick et al., 2014). However, additional studies are needed to explore novel biomarkers to predict the prognosis of glioma patients.

N6-methyladenosine (m6A) methylation is the most common type of RNA modification that mainly occurs in the messenger RNA (mRNA) of eukaryotes (Huisman et al., 2017). The dynamic modification of m6A is regulated by “writers” (methyltransferases), “readers” (binding proteins), and “erasers” (demethylases) (Yang et al., 2018). The biological functions of m6A are mediated by the “readers” that specifically recognize the methylated adenosine on mRNA. The “writers” mainly include methyltransferase like (METTL) family (*METTL3*, *METTL5*, *METTL14*, and *METTL16*), *KIAA1429*, *WTAP*, RNA-binding motif (RBM) family (*RBM15* and *RBM15B*), and *ZC3H13*, which stimulate the methylation of m6A on RNA (Meyer and Jaffrey, 2017; Wang et al., 2017). The “readers” are composed of YTH domain-containing (YTHDC) family (*YTHDC1* and *YTHDC2*), YTH domain family (YTHDF) family (*YTHDF1*, *YTHDF2*, and *YTHDF3*), *HNRNPC*, *FMR1*, *EIF3A*, and insulin-like growth factor-2 mRNA-binding proteins (IGF2BP) family (*IGF2BP1*, *IGF2BP2*, and *IGF2BP3*) (Zaccara et al., 2019). Besides, the “erasers” comprise fat mass and obesity-associated protein (FTO) and alkB homolog 5 (ALKBH5) (Jia et al., 2011; Zheng et al., 2013). Various studies have revealed that m6A regulators can be used as novel prognostic biomarkers in different types of cancer (Chen et al., 2019; Fang and Chen, 2020; Huang et al., 2020a; Li Z. et al., 2020; Lu et al., 2020). *METTL3* was shown to promote the proliferation and migration of hepatocellular carcinoma via the *YTHDF2*-dependent pathway and its knockdown could inhibit tumor progression (Chen et al., 2018). Moreover, *YTHDF1* was found to be highly expressed in colorectal cancer and the knockdown of *YTHDF1* could significantly suppress the tumor growth both *in vitro* and *in vivo* (Bai et al., 2019). Additionally, the low expression of *FTO* was found to indicate poor prognosis in patients with intrahepatic cholangiocarcinoma (Rong et al., 2019). These findings indicate that m6A regulators are highly involved in cancer development with promising prognostic values.

In recent years, numerous studies have proved that tumor immune microenvironment plays a crucial role in cancer progression and therapeutic efficacy (Quail and Joyce, 2017). The brain was long considered to be an “immune privileged” organ. However, this viewpoint was challenged when the lymphatic vessels were discovered along with the dural sinuses in mice (Louveau et al., 2015). Immune cells could infiltrate into the brain and form the immune microenvironment with other components. Multiple studies have identified the immunosuppressive status in gliomas, in which tumor-associated macrophages and regulatory T (Treg) cells in glioma microenvironment suppress the activities of T cells, mediating the immune escape of gliomas (Colombo and Piconese, 2007). Besides, glioma cells increase the expression of immunosuppressive factors such as programmed cell death 1 ligand (PD-L1) to reduce the presentation of antigens (Bloch et al., 2013; Xu et al., 2020). Therefore, the content of the immune microenvironment was closely associated with the efficacy of immunotherapy.

A previous study has revealed that m6A regulators are associated with glioma progression and prognosis (Chai et al., 2019). However, the relationship between m6A regulators and the immune microenvironment in gliomas remains unclear. In this study, we extracted data from the Chinese Glioma Genome Atlas (CGGA), The Cancer Genome Atlas (TCGA), and the Rembrandt datasets to explore the expression pattern and prognostic value of m6A regulators in gliomas. Besides, clustering subgroups and risk models were established based on the expression of m6A regulators to validate the predictive value of m6A regulators in risk stratification and prognosis. Moreover, the association between m6A regulators and the immune microenvironment was explored using the constructed signature. Additionally, the predictive value of m6A signature in the efficacy of chemotherapy and radiotherapy was also investigated. Our study aims to comprehensively assess the correlation of m6A regulators with prognosis, immune microenvironment, and therapeutic efficacy in gliomas.

## Materials and Methods

### Data Extraction

All RNA-seq data and clinical characteristics of enrolled samples were extracted from CGGA^1^, TCGA^2^, and Rembrandt databases. A total of 2144 glioma samples were enrolled in our study, in which 325 samples extracted from the CGGA database (CGGA325) were defined as the training cohort; 693 samples extracted from the CGGA database (CGGA693), 651 samples extracted from the TCGA database, and 475 samples extracted from the Rembrandt database were defined as the validation cohort; 20 normal tissues extracted from the CGGA database were termed as the control group. Data of CGGA325 and CGGA693 datasets were obtained in fragments per kilobase of exon model per million mapped fragments (FPKM) format. Data of TCGA dataset were batch normalized counts format, whereas that of Rembrandt dataset was normalized microarray format. The characteristics of patients in the training and validation cohorts were summarized in Table 1.

**TABLE 1:** Characteristics of patients in training cohort and validation cohorts.

**Features**	**Training cohort**	**Validation cohort**		
	**CGGA325 (*n* = 325)**	**CGGA693 (*n* = 693)**	**TCGA (*n* = 651)**	**Rembrandt (*n* = 475)**
**Age**				
≤41	150	322	280	161
>41	175	370	371	233
NA	0	1	0	81
**Gender**				
Male	203	398	375	203
Female	122	295	276	121
NA	0	0	0	151
**Histology**				
A	56	119	63	85
O	52	69	186	37
AA	62	152	131	47
AO	12	103	134	20
GBM	139	249	136	183
NA	4	1	1	103
**Grade**				
I	0	0	0	2
II	103	188	249	92
III	79	255	265	70
IV	139	249	136	181
NA	4	1	1	130
**IDH status**				
Mutant	175	356	428	NA
Wild-type	149	286	216	NA
NA	1	51	7	NA
1p/19q status				
Codel	67	145	169	24
Non-codel	250	478	478	150
NA	8	70	4	301
**Chemotherapy**				
Yes	178	457	NA	NA
No	124	151	NA	NA
NA	23	85	NA	NA
**Radiotherapy**				
Yes	258	509	NA	NA
No	51	113	NA	NA
NA	16	71	NA	NA

*A, astrocytoma; O, oligodendroglioma/oligoastrocytoma; AA, anaplastic astrocytoma; AO, anaplastic oligodendroglioma/oligoastrocytoma; GBM, glioblastoma multiforme; IDH, isocitrate dehydrogenase.*

### Identification of m6A Regulators

A total of 22 m6A regulators were identified according to previous studies (Wang et al., 2017; Yang et al., 2018; Yi et al., 2020). The expression level of these genes was assessed between glioma samples and normal pairs.

### Consensus Clustering

The consensus clustering was performed using “ConsensusClusterPlus” R package to categorize patients with gliomas into subgroups. The clustering algorithm was partitioning around medoids and the distance was measured by the euclidean metric.

### Stroma and Immune Scores Calculation

The stroma and immune scores were measured by Estimation of Stromal and Immune cells in Malignant Tumor tissues using Expression data (ESTIMATE) analysis using “estimate” R package (Yoshihara et al., 2013). Tumor purity was calculated according to the algorithm.

### Immune Cells Infiltration Analysis

The abundance of six immune cells including B cells, CD4+ T cells, CD8+ T cells, neutrophils, macrophages, and dendritic cells was calculated by Tumor Immune Estimation Resource (TIMER) algorithm (Li et al., 2017). The role of copy number alternations (CNAs) of m6A regulators on immune cell infiltration was evaluated using TIMER algorithm^3^.

### Construction of Risk Signature

The selection of candidate risk m6A regulators was performed by the least absolute shrinkage and selection operator (LASSO) analysis. Multivariate Cox regression analysis was used to profile independent prognostic genes. Variance inflation factor (Vif) and hypothesis testing were used to filter out genes with high collinearity. Risk score for each patient in the training and validation cohort was calculated by the following algorithm: Risk score = 0.052 × *YTHDF2* + 0.025 × *ALKBH5* + 0.029 × *KIAA1429* + 0.023 × *IGF2BP3*. The patients were divided into high risk and low risk groups based on the mean value of the risk score.

### Statistical Analysis

Statistical analyses and visualization were mainly performed using R version 4.0.2, GraphPad Prism version 8.0.1, and TBtools (Chen et al., 2020). Kaplan–Meier and log-rank analysis were used to evaluate the survival differences between grouped patients. Subgroup analysis was used to assess the stability of the risk signature, in which patients were divided into two subgroups based on their age (≤41 and >41 years old) and gender (female and male). Time-dependent receiver operating characteristic (ROC) curve analysis was used to evaluate the predictive value of constructed risk model using “survivalROC” R package. Student’s *t*-test and one-way ANOVA analysis were used to estimate the differences between two groups and more than two groups. The correlation of gene expression was calculated using “spearman” method. Two-sided *p* < 0.05 was regarded as statistically significant.

## Results

### Expression of m6A Regulators Was Involved in the Progression and Development of Gliomas

**FIGURE 1:**
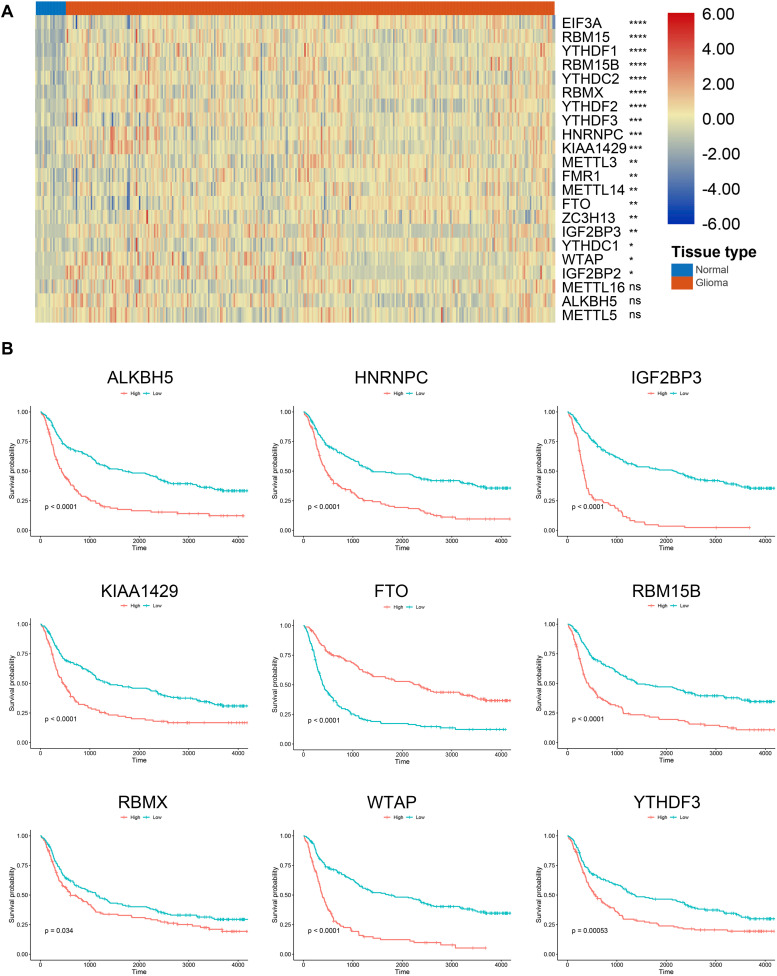
Expression and survival analysis of m6A regulators in gliomas. **(A)** Expression of m6A regulators in glioma and normal tissues. **(B)** Survival analysis of m6A regulators in gliomas. *p < 0.05; **p < 0.01; ***p < 0.001; ****p < 0.0001; ns, no significance.

According to previous studies, we selected 22 m6A regulators for further investigation. The RNA expression of these genes was extracted from the training cohort. Results showed that 19 m6A regulators were significantly higher in gliomas compared with normal brain tissue (*p* < 0.05) (Figure 1A); whereas no significant difference was detected between gliomas and normal tissue regarding the expression of *METTL16*, *ALKBH5*, and *METTL5* (*p* > 0.05). Besides, Kaplan–Meier analysis revealed that 15 m6A regulators were independent prognostic genes and the other regulators including *YTHDC1, FMR1, ZC3H13, METTL14, EIF3A, METTL3, METTL16*, and *IGF2BP1* were not associated the prognosis of glioma patients. Generally, patients with low expression of m6A regulators enjoyed favorable prognoses (*p* < 0.05); whereas patients with low expression of *FTO* had a less survival time compared to those with high expression of *FTO* (*p* < 0.05) (Figure 1B). These results indicated that m6A regulators were highly involved in the progression and development of gliomas.

### Expression of m6A Regulators Was Associated With Current Glioma Prognostic Markers

**FIGURE 2:**
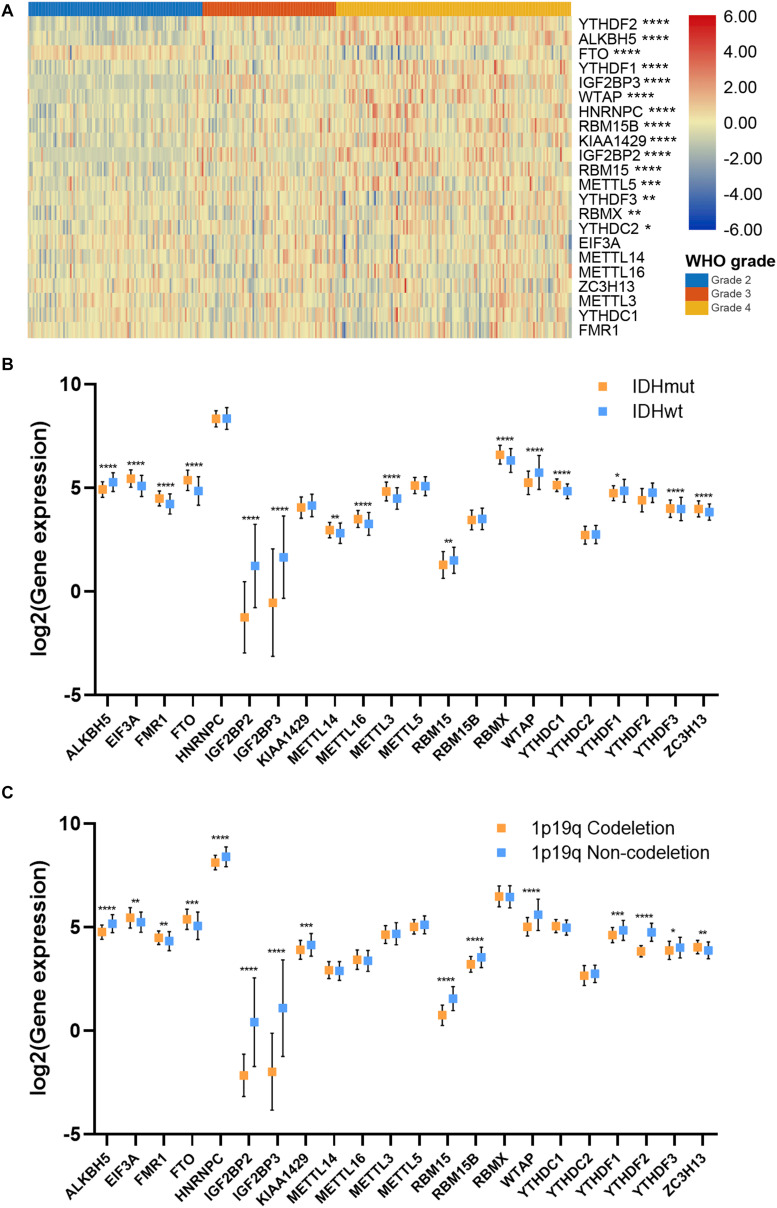
Association between m6A regulator expressions and glioma features. **(A)** Expression pattern of m6A regulators in different grades of glioma. **(B)** Expression of m6A regulators in IDH mutant and IDH wild-type gliomas. **(C)** Expression of m6A regulators in 1p19q codeletion and 1p19q non-codeletion gliomas. IDH, isocitrate dehydrogenase. *p < 0.05; **p < 0.01; ***p < 0.001; ****p < 0.0001.

To further investigate the role of m6A regulators in gliomas, we explored the expression of m6A regulators in different subgroups of gliomas. Results showed that the expression of 15 m6A regulators, which had independent prognostic value, was significantly different in different grades of gliomas (Figure 2A). Generally, the high expression of m6A regulators indicated a higher grade of glioma; whereas the high expression of *FTO* indicated a lower grade of glioma. Additionally, the expression of 16 m6A regulators was significantly different in IDH mutant and IDH wild-type gliomas (*p* < 0.05), in which 6 genes were down-regulated and 10 genes were up-regulated in IDH mutant gliomas compared with IDH wild-type gliomas (Figure 2B). Moreover, the expression of 14 m6A regulators in 1p19q codeletion and non-codeletion gliomas was evident (*p* < 0.05), in which 11 genes were down-regulated and 3 genes were up-regulated in 1p19q codeletion gliomas compared with non-codeletion gliomas (Figure 2C). A total of 11 m6A regulators had a consistent expression pattern in IDH and 1p19q subgroups, in which four genes were up-regulated (*FTO, FMR1, EIF3A*, and *ZC3H13*) and seven genes were down-regulated (*ALKBH5, IGF2BP2, IGF2BP3, RBM15, WTAP, YTHDF1*, and *YTHDF3*) in IDH mutant and 1p19q codeletion gliomas. Given that patients with the lower grade, IDH mutant, and 1p19q codeletion gliomas had better prognoses, our findings suggested that m6A regulators had promising values in predicting the prognosis of glioma patients.

### Consensus Clustering for m6A Regulators Correlated With Glioma Prognosis and Immune Microenvironment

**FIGURE 3:**
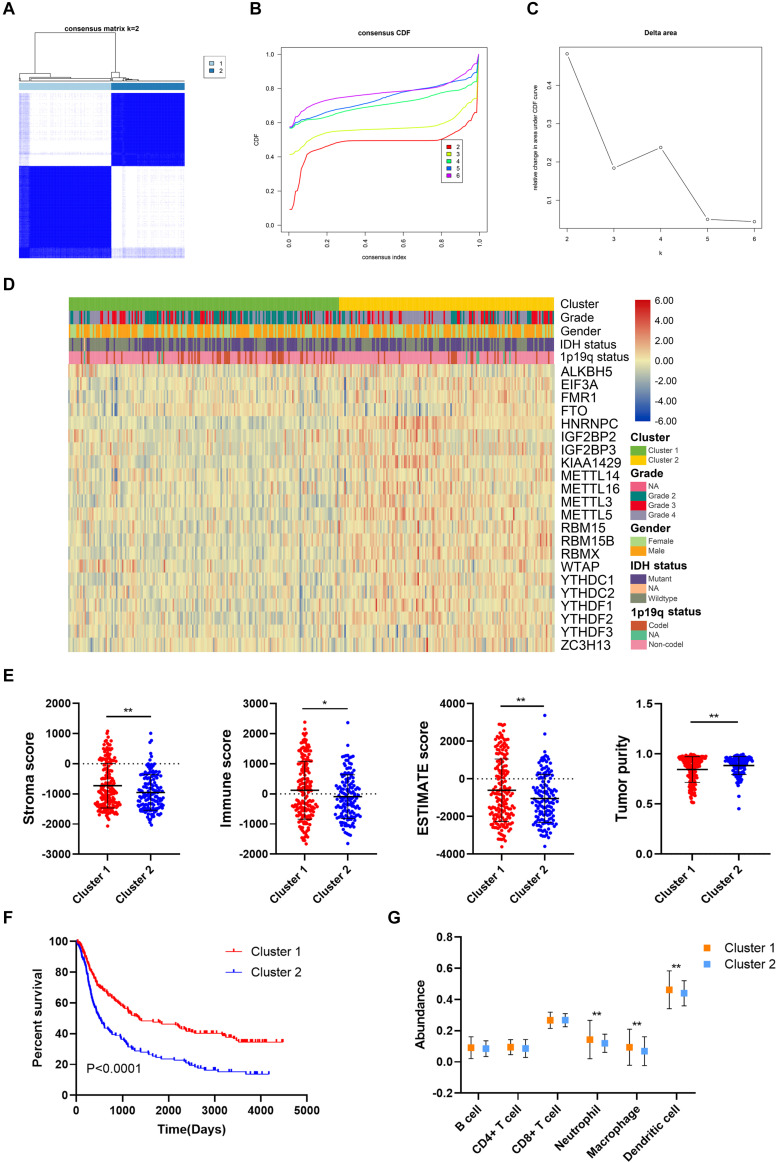
Prognosis and immune infiltrations in consensus clustering subgroups of gliomas. **(A)** Consensus clustering matrix for *k* = 2. **(B,C)** Consensus clustering cumulative distribution function for *k* = 2–6. **(D)** Expression pattern of m6A regulators in cluster 1 and cluster 2 subgroups. **(E)** Stroma, immune, and ESTIMATE scores and tumor purity in cluster 1 and cluster 2 subgroups. **(F)** Kaplan–Meier analysis of patients in cluster 1 and cluster 2 subgroups. **(G)** The abundance of six immune cells in cluster 1 and cluster 2 subgroups. *p < 0.05; **p < 0.01.

The unsupervised clustering method, consensus clustering, was performed to classify patients in the training cohort into subgroups based on the expression of m6A regulators. *K* = 2 was identified with optimal clustering stability (Figure 3A–C). A total of 325 patients in the training cohort were clustered into two subgroups, 181 patients in cluster 1 and 144 patients in cluster 2. The expression pattern of m6A regulators in cluster 1 and cluster 2 was shown by the heatmap (Figure 3D). The expression level of m6A regulators (except *FTO*) were lower in cluster 1 compared with cluster 2. Besides, the stroma (*p* < 0.05), immune (*p* < 0.05), and ESTIMATE scores (*p* < 0.05) were significantly higher whereas the tumor purity was markedly lower (*p* < 0.05) in cluster 1 compared with cluster 2 (Figure 3E). Moreover, the overall survival of patients in cluster 1 was significantly longer than those in cluster 2 (*p* < 0.05) (Figure 3F). Additionally, the abundance of neutrophil, macrophage, and dendritic cell was significantly higher in cluster 1 compared with cluster 2 (*p* < 0.05), whereas no difference was detected regarding B cell, CD4+ T cell, and CD8+ T cell (Figure 3G). These results indicated that the clustering subgroups based on m6A regulators were closely related to prognosis and the immune microenvironment in gliomas.

### Construction of Risk Signature Based on m6A Regulators Expression in the Training Cohort

**FIGURE 4:**
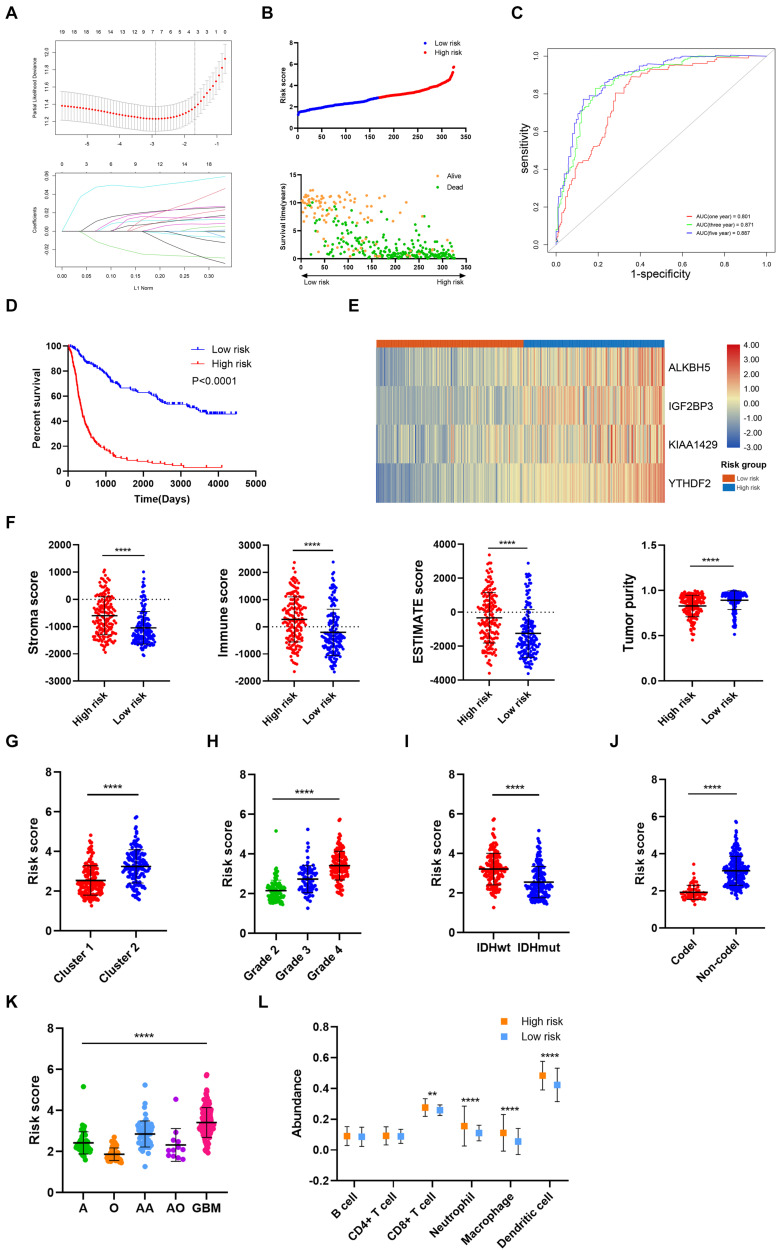
Construction and analysis of risk signature based on m6A regulator expression in the training cohort. **(A)** LASSO analysis with minimal lambda value. **(B)** Risk score of each patient in the training cohort. **(C)** Time-dependent ROC analysis of risk score in predicting prognoses. **(D)** Kaplan–Meier analysis of patients in the high risk and low risk groups. **(E)** Expression pattern of four candidate m6A regulators in the high risk and low risk groups. **(F)** Stroma, immune, and ESTIMATE scores and tumor purity in the high risk and low risk groups. **(G)** Risk score of cluster 1 and cluster 2 subgroups. **(H)** Risk score of different grades of glioma. **(I)** Risk score of IDH wild-type and IDH mutant gliomas. **(J)** Risk score of 1p19q codeletion and non-codeletion gliomas. **(K)** Risk score of different histological subtypes of glioma. **(L)** The abundance of six immune cells in the high risk and low risk groups. ROC, receiver operating characteristic; IDH, isocitrate dehydrogenase. **p < 0.01; ****p < 0.0001.

Then, the risk signature was established to evaluate the predictive value of m6A regulators. LASSO analysis filtered out seven m6A regulators with the minimum lambda value (Figure 4A). After hypothesis testing, four m6A regulators including *ALKBH5*, *IGF2BP3*, *KIAA1429*, and *YTHDF2* were selected with *p*-value of less than 0.05 and Vif of less than 2. The risk signature was constructed by multivariate Cox analysis (Supplementary Figure 1). Patients in the training cohort were divided into high risk and low risk groups based on risk scores (Figure 4B). Multivariate Cox analysis revealed that risk score was the independent risk factor for patients in the training cohort (Table 2). Time-dependent ROC analysis revealed that the predictive accuracy of risk score was highest in predicting 5-year survival (Figure 4C). The area under curve (AUC) of 1-year, 3-year, and 5-year survival was 0.801, 0.871, and 0.887, respectively. Moreover, patients in the low risk group had a longer overall survival compared with those in the high risk group (*p* < 0.05) (Figure 4D); when patients were divided into subgroups based on their age and gender, those in the low risk group still had a longer survival time (*p* < 0.05) (Supplementary Figure 2A). The expression of four candidate m6A regulators was higher in high risk group compared with the low risk group (Figure 4E). The stroma, immune, and ESTIMATE scores were significantly higher (*p* < 0.05) whereas tumor purity was lower in the high risk group compared with the low risk group (*p* < 0.05) (Figure 4F). In cluster 1, which had a better prognosis, the risk score was notably lower than cluster 2 (*p* < 0.05) (Figure 4G). Besides, the risk score was elevated in the higher grade, IDH wild-type, and 1p19q non-codeletion subtype of glioma (*p* < 0.05) (Figures 4H–J). As for the histological subtype of glioma, the risk score was increased in the relatively malignant glioma (e.g., anaplastic astrocytoma vs astrocytoma; anaplastic oligodendroglioma/oligoastrocytoma vs oligodendroglioma/oligoastrocytoma); the glioblastoma, which was the most malignant glioma, had the highest risk score (Figure 4K). Moreover, the abundance of CD8+ T cell, neutrophil, macrophage, and dendritic cell was significantly higher in the high risk group compared with the low risk group (*p* < 0.05) (Figure 4L). These results indicated that the constructed risk signature exhibited a potent value in predicting the prognosis of glioma patients. The risk score was closely related with the immune microenvironment in gliomas.

**TABLE 2:** Multivariate analyses of risk score and clinical features in training cohort.

**Variables**	**Coefficient**	**HR**	**HR 95%CI (lower)**	**HR 95%CI (upper)**	***p*-Value**
Risk score	0.583	1.792	1.437	2.235	<0.0001
Histology	–0.399	0.671	0.532	0.847	0.001
Grade	1.196	3.308	2.214	4.943	<0.0001
Gender	0.052	1.053	0.792	1.401	0.720
Age	0.011	1.011	0.998	1.025	0.091
Radiotherapy	–0.522	0.593	0.418	0.842	0.003
Chemotherapy	–0.364	0.695	0.509	0.949	0.022
IDH status	0.055	1.057	0.754	1.481	0.748
1p19q status	0.001	1.001	0.520	1.924	0.999

*HR, hazard ratio; CI, confidence interval; IDH, isocitrate dehydrogenase.*

### Risk Signature Was Associated With Glioma Prognosis and Immune Microenvironment in the Validation Cohort

**FIGURE 5:**
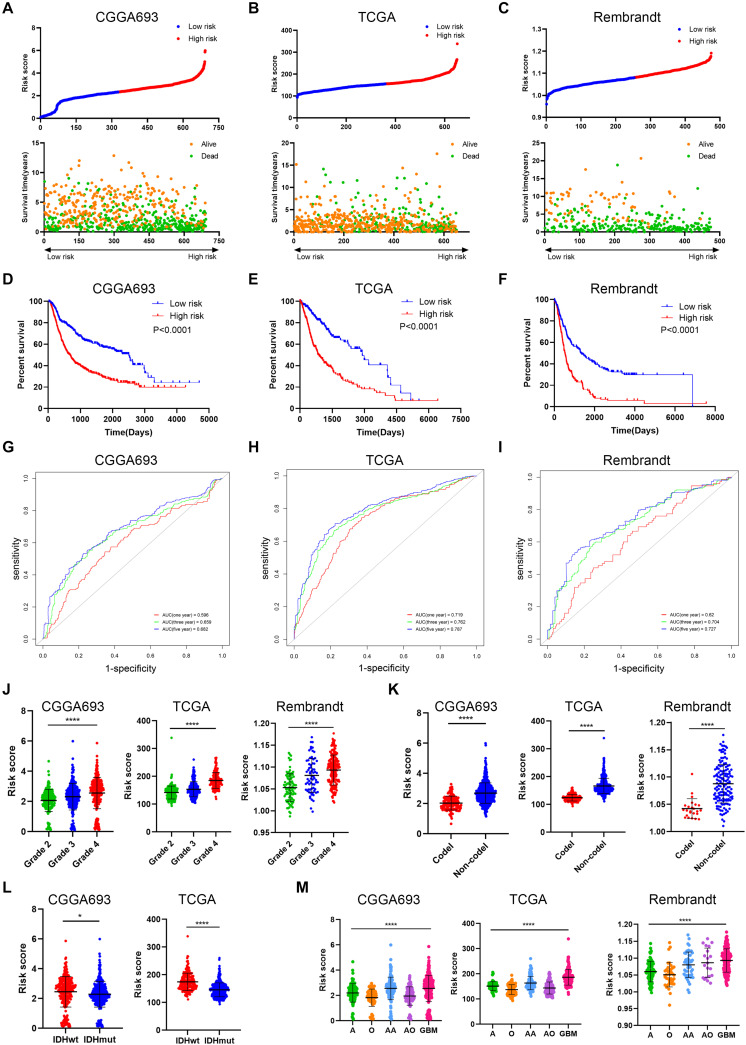
Verification of risk signature of glioma features in the validation cohort. **(A–C)** Risk score of each patient in the three datasets. **(D–F)** Kaplan–Meier analysis of patients in the high risk and low risk groups in the three datasets. **(G–I)** Time-dependent ROC analysis of risk score in predicting prognoses in the three datasets. **(J)** Risk score of different grades of glioma in the three datasets. **(K)** Risk score of 1p19q codeletion and non-codeletion gliomas in the three datasets. **(L)** Risk score of IDH wild-type and IDH mutant gliomas in CGGA693 and TCGA datasets. **(M)** Risk score of different histological subtypes of glioma in the three datasets. ROC, receiver operating characteristic. *p < 0.05; ****p < 0.0001.

To further verify the predictive value of risk signature, we applied the risk score algorithm in the validation cohort. The risk score of patients in the validation cohort was calculated. All patients were divided into high risk and low risk groups based on the mean value of risk score (Figures 5A–C). Multivariate Cox analysis revealed that risk score was the independent risk factor for patients in the validation cohort (Supplementary Table 1). Besides, Kaplan–Meier analysis revealed that patients in the low risk group had a better prognosis compared with those in the high risk group (*p* < 0.05) (Figures 5D–F); when patients were divided into subgroups based on their age and gender, those in the low risk group still had a better prognosis (*p* < 0.05) (Supplementary Figure 2B–D). Time-dependent ROC analysis showed that predictive accuracy of risk score was highest in predicting long-term survival (Figures 5G–I). The AUC of 1-year, 3-year, and 5-year survival in CGGA693 dataset was 0.596, 0.659, and 0.682, respectively; that in TCGA dataset was 0.719, 0.762, and 0.787, respectively; that in Rembrandt dataset was 0.620, 0.704, and 0.727, respectively. Besides, the risk score was elevated in the high-grade, IDH wild-type, and 1p19q non-codeletion subtype of glioma in the validation cohort (*p* < 0.05), which was consistent with the training cohort (Figures 5J–L). Moreover, the risk score was relatively high in the malignant subtype of glioma and glioblastoma had the highest risk score, which was consistent with the training cohort (*p* < 0.05) (Figure 5M).

**FIGURE 6:**
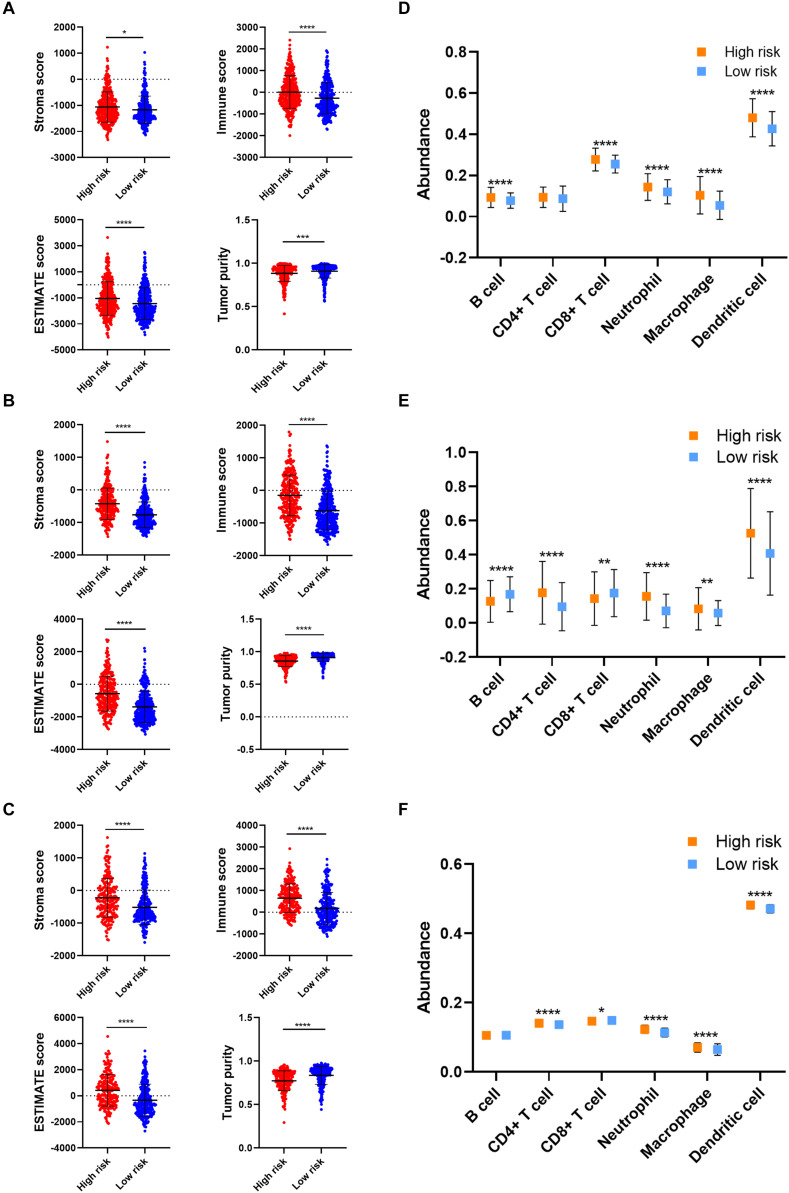
Verification of risk signature of glioma immune microenvironment in the validation cohort. **(A–C)** Stroma, immune, and ESTIMATE scores and tumor purity in the high risk and low risk groups in CGGA693 **(A)**, TCGA **(B)**, and Rembrandt **(C)** datasets. **(D–F)** The abundance of six immune cells in the high risk and low risk groups in CGGA693 **(D)**, TCGA **(E)**, and Rembrandt **(F)** datasets. *p < 0.05; **p < 0.01; ***p < 0.001; ****p < 0.0001.

As for the immune microenvironment of gliomas, the stroma, immune, and ESTIMATE scores were significantly higher whereas the tumor purity was lower in the high risk group compared with the low risk group in the validation cohort (*p* < 0.05) (Figures 6A–C), which was consistent with the training cohort. Moreover, the abundance of CD8+ T cell, neutrophil, macrophage, and dendritic cell was significantly higher in the high risk group compared with the low risk group in the validation cohort (*p* < 0.05), which was consistent with the training cohort (Figures 6D–F). These results confirmed the establishment of the risk signature and its association with the prognosis and immune microenvironment of gliomas.

Additionally, the effect of CNAs of four candidate m6A regulators in risk signature on immune cell infiltration was further investigated to provide a novel insight into the association between risk score and immune cell infiltration. The arm-level gain of *ALKBH5* and *IGF2BP3* was closely associated with the infiltration of six types of immune cells (Supplementary Figures 3A,B); whereas CNAs of *KIAA1429* did not exhibit an association with immune cell infiltration (Supplementary Figure 3C). Besides, the high amplication and arm-level deletion of *YTHDF2* were significantly related to immune cell infiltration (Supplementary Figure 3D). These results suggested that the four candidate genes of the risk signature were closely associated with the infiltration of immune cells.

### Risk Stratification Was Associated With the Efficacy of Chemoradiotherapy and Immunotherapy

**FIGURE 7:**
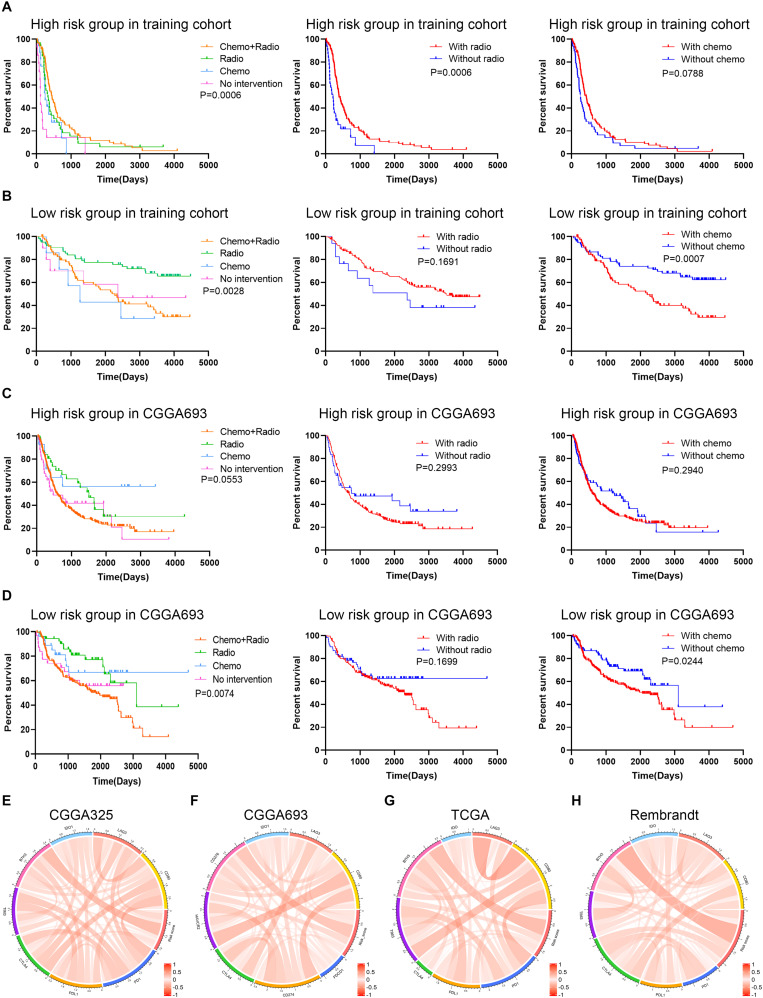
Exploration of the association between risk signature and therapeutic efficacy. **(A,B)** Kaplan–Meier analysis of therapeutic efficacy of patients in the high risk and low risk groups in the training cohort. **(C,D)** Kaplan–Meier analysis of therapeutic efficacy of patients in the high risk and low risk groups in the validation cohort. **(E–H)** The correlation between risk score and immune checkpoints expression in CGGA325 **(E)**, CGGA693 **(F)**, TCGA **(G)**, and Rembrandt **(H)** datasets.

The association between therapeutic efficacy and risk stratification was also explored. In the training cohort, patients in the high risk group who received chemoradiotherapy had longer survival compared with those receiving other therapies (*p* = 0.0006), and radiotherapy exhibited a potent efficacy (*p* = 0.0006) (Figure 7A); patients in the low risk group who received single radiotherapy had better prognoses compared with those without radiotherapy (*p* = 0.0028), and chemotherapy did harm to the survival rates (*p* = 0.0007) (Figure 7B). In the validation cohort, monotherapy of chemotherapy and radiotherapy had no significant effect on improving the survival rate of patients in the high risk group (Figure 7C); however, patients in the low risk group who received single chemotherapy or radiotherapy had better prognoses compared with those receiving other therapies (*p* = 0.0074), and chemotherapy decreased the survival rates (*p* = 0.0244) (Figure 7D). Besides, the risk score was correlated with the expression of immune checkpoints (i.e., *PD-1*, *PD-L1*, *CTLA-4*, and *B7H3*) in the training and validation cohorts, in which the risk score had a notable relationship with the expression of B7H3 (Figures 7E–H). These results showed that for patients in the low risk group, radiotherapy exhibited a potent efficacy whereas chemotherapy even decreased the survival rates. Therefore, the risk stratification was associated with the efficacy of chemoradiotherapy and might predict the efficacy of immunotherapy in gliomas.

## Discussion

According to WHO grade classification of gliomas, glioma was categorized into four grades, in which a higher grade indicated a worse survival. The past 30 years of research into glioma biology led to the discovery of hundreds of molecular alterations in grade II, III, and IV gliomas. Molecular exploration is in need of improved outcomes and the value of prognosis. Turkalp et al. (2014) found that IDH1/2, as an early event in the development of glioma, provided some reference in predicting glioma. Akagi et al. (2018) also showed that oligodendroglioma patients with IDH-mutant and 1p/19q co-deleted, rather than the WHO grade, demonstrated a better overall survival.

The previous studies inspired us to explore the changes in the expression of other genes and proteins in relation to the occurrence and development of glioma and to guide our intensive study on the role of glioma genes. With the booming development of high-throughput sequencing, various databases about genomic profiles have been established by researchers, which makes us to clearly acknowledge the genomic changes. Previous studies have revealed that immune-related gene signature was associated with prognosis and immune infiltration in gliomas (Qin et al., 2020; Zhang et al., 2020). Their risk scores exhibited potent predictive values in the diagnosis and prognosis of gliomas. Huang et al. (2020c) found that ATP metabolism-related signature was associated with prognosis and immune microenvironment in gliomas. Besides, the risk score had a potent predictive accuracy identifying mesenchymal subtype glioma. Tao et al. (2020) revealed that epithelial-mesenchymal transition (EMT) based signature was also correlated with prognosis and clinicopathological features of glioma. However, few research explored the signature based on m6A regulators in gliomas.

Our study would fill the blank of m6A-related signature in the prediction of gliomas. The m6A regulator-based signature was closely associated with the immune microenvironment of glioma. Consensus clustering based on m6A regulator expressions was performed to divide the training cohort into two clusters. Survival analysis revealed that patients in cluster 1 had a favorable survival. Besides, the immune and stroma scores and immune cell infiltration was higher in cluster1. However, previous studies revealed that high immune and stroma scores as well as high infiltration of macrophages were associated with poor prognosis, which was reversed with our results in consensus clustering (Deng et al., 2020; Ni et al., 2020; Tian et al., 2020). Therefore, we further constructed a risk model to explore and validate the association between m6A regulators and the immune microenvironment. This study constructed the risk signature based on the expression of m6A regulators and also revealed that risk score was associated with prognosis and immune infiltration, which was similar to other studies. The risk stratification based on risk signature might be used to predict the efficacy of chemoradiotherapy and immunotherapy.

The risk signature correlates with glioma types, instead of other factors such as age and gender. In different histological subtypes of glioma, the risk score had a elevated tendency in the relatively malignant subtype. For example, the risk score was significantly higher in anaplastic astrocytoma compared to astrocytoma. Although the risk score of anaplastic oligodendroglioma or oligoastrocytoma was not significantly higher than astrocytoma, it is notably higher than oligodendroglioma or oligoastrocytoma. Moreover, the most malignant glioma, glioblastoma, had the highest risk score. Therefore, the risk score was closely associated with the histological subtype of glioma. In order to exclude the interference of other factors such as age and gender, we conducted subgroup analysis to further validate the predictive value of risk score. Results showed that patients with high risk score in the age and gender subgroups still had poor prognoses in four datasets. Besides, multivariate Cox analysis confirmed that the risk score was independent risk factor for glioma patients in four datasets. These results suggested that the risk signature was not affected by other factors such as age and gender.

This risk signature in compliance with current molecular pathology based survival prediction. IDH mutation, MGMT promoter methylation, and 1p19q codeletion in gliomas were associated with more favorable prognoses. A total of 11 m6A regulators had similar expression patterns in IDH and 1p19q subgroups, in which four were up-regulated and seven were down-regulated in IDH mutant and 1p19q codeletion gliomas. Unexpectedly, *FMR1, EIF3A*, and *ZC3H13*, whose high expressions indicated poor prognosis, were up-regulated in the IDH mutant and 1p19q codeletion gliomas since the two genomic mutations indicated favorable prognoses. However, IDH mutant and 1p19q codeletion only stand for a large proportion of patients with relatively good prognoses but not absolutely all patients have longer survival time. Therefore, those genes up-regulated in the IDH mutant and 1p19q codeletion subgroups may indicate worse prognoses within the subgroup. By the combination of the risk score and current indicators, we can predict the prognosis of glioma patients more accurately and efficiently.

The risk signature was established in multiple types of cancers with potent predictive values (Huang et al., 2020d; Wang et al., 2020; Zhang et al., 2020). On one hand, the risk score was calculated based on the coefficient and expression of candidate genes. Typically, a higher risk score indicated a poor survival. In our study, four candidate genes (*ALKBH5*, *IGF2BP3*, *KIAA1429*, and *YTHDF2*) were selected to construct the risk signature. The risk score exhibited promising value in predicting 5-year survival in the training and validation cohorts (AUC = 0.887, 0.682, 0.787, and 0.727). Although the predictive value in CGGA693 dataset was relatively low, its value was further validated by the other two datasets. Therefore, we believed that the risk score could predict long-term survival with a relatively high accuracy. On the other, the immune and stroma scores, as well as immune cell infiltration, were significantly higher in the high risk group. Moreover, the risk score was notably lower in cluster 1 compared with cluster 2, indicating the risk signature could replace the consensus clustering with a better evaluation efficiency. Further, the constructed risk signature was verified in the validation cohort, which exhibited consistent results with the training cohort. Four candidate genes were highly expressed in the high risk group and exhibited as risk factors for glioma patients. *ALKBH5* was found to play an oncogenic role in epithelial ovarian cancer (Zhu et al., 2019). Besides, the inhibition of *ALKBH5* could reduce the proliferation of glioblastoma stem-like cells via *FOXM1* axis (Zhang et al., 2017). Previous studies revealed that *IGF2BP3* promoted tumor proliferation and progression in colorectal cancer, gastric cancer, and glioblastoma (Suvasini et al., 2011; Zhou et al., 2017; Xu et al., 2019). In the end, *KIAA1429* was proved to facilitate cancer progression in breast and live cancer (Lan et al., 2019; Qian et al., 2019). Similarly, *YTHDF2* was shown to be involved in the progression and angiogenesis in hepatocellular carcinoma, and its inhibition exhibited promising efficacy in acute myeloid leukemia (Chen et al., 2018; Li et al., 2018; Hou et al., 2019; Paris et al., 2019). These findings revealed that the dysregulation of candidate m6A regulators was involved in the tumor progression in various kinds of cancer.

The low expression of *FTO* were associated with poor prognosis in gliomas, although there have been a contentious and divisive. Among 22 m6A regulators, 19 were highly expressed in glioma tissues compared with normal tissues, indicating that these m6A regulators might play crucial roles in gliomas. Survival analysis revealed that 15 m6A regulators were identified as prognostic indicators, in which the high expression of 14 m6A regulators and the low expression of *FTO* were associated with poor prognosis, whereas the high expression of *FTO* indicated a favorable prognosis. These results suggested that although *FTO* was highly expressed in glioma tissues, it might play a protective role for patients with gliomas. Li J. et al. (2020) found that the expression of m6A regulators was dysregulated in osteosarcoma, and low expression of *FTO* and high expression of *YTHDF3* was associated with poor prognosis, which was consistent with our findings. Besides, *FTO* could suppress the self-renewal of ovarian cancer stem cells via inhibiting cyclic adenosine monophosphate (cAMP) signaling pathway (Huang et al., 2020b). However, Su et al. (2020) revealed that the inhibition of *FTO* also exhibited potent anti-tumor effects in multiple types of cancers. *FTO* inhibition enhanced the cytotoxicity of T cell and reduced immune evasion by suppressing the expression of immune checkpoint genes. These findings suggested that *FTO* played a complicated role in different types of cancer, and its role in glioma needed further investigation.

Those m6A regulators were involved in the immune microenvironment of gliomas. When patients in the training and validation cohorts were divided into high risk and low risk groups based on the risk score, the high risk group had a higher stroma and immune scores and a lower tumor purity. Previous studies found that stroma and immune scores calculated by ESTIMATE algorithm were meaningful for the classification and prognosis of glioblastoma and lower-grade gliomas (Jia et al., 2018; Deng et al., 2020). Besides, the abundance of immune cells such as CD8 + T cell, neutrophil, macrophage, and dendritic cell was highly infiltrated in the high risk group. Further, we identified that CNAs (i.e., arm-level gain, arm-level deletion, and high amplication) of four candidate genes were markedly associated with immune cell infiltrations.

The risk stratification could facilitate the determination of therapeutic options to improve prognoses. Current management for gliomas includes surgery, chemotherapy, and radiotherapy, which cannot reach optimal remission although great advancement has been achieved. In our study, patients in the high risk group who received chemoradiotherapy exhibited the most favorable survival, whereas radiotherapy showed great efficacy. However, this result was not consistent in the validation cohort. Notably, patients in the low risk group who received chemoradiotherapy showed worse prognosis compared with those receiving chemotherapy or radiotherapy. In contrast, single radiotherapy exhibited promising efficacy in the training and validation cohorts although radiotherapy had no effect in improving the survival rates of patients. However, it should be noted that a part of patients with or without radiotherapy would receive chemotherapy, which was demonstrated to impair the survival rate of patients. Therefore, single radiotherapy should be considered for patients in the low risk group. Although patients received single chemotherapy exhibited favorable prognosis in the validation cohort, the finding was not consistent with the training cohort. Since chemotherapy was found to impair the survival rates of patients with low risk in training and validation cohorts, chemotherapy should be deliberated for patients in the low risk group. Additionally, the risk score was positively correlated with the expression of B7H3. The novel chimeric antigen receptor T (CAR-T) product targeting B7H3 exhibited promising efficacy in the treatment of glioblastoma both *in vitro* and *in vivo*. Currently, the clinical studies were undergoing to evaluate the efficacy of B7H3 CAR-T cell in the treatment of glioblastoma (NCT04077866) and pediatric gliomas (NCT04185038) (Xu et al., 2020). The risk stratification based on the risk score might help predict the efficacy of B7H3 CAR-T therapy.

There are still limitations in our study. Firstly, our findings are based on open accessed databases without our cohort. Secondly, the interactions between m6A regulators and immune cells were not validated by experiments. Last but not least, the regulatory mechanism of m6A regulators in glioma immune microenvironment is not elucidated, which warrants further investigation to provide a better understanding.

## Conclusion

To sum up, our study comprehensively assessed the expression pattern and prognostic value of m6A regulators in gliomas. The expression of m6A regulators was associated with the classification of glioma subtypes. Besides, the consensus clustering and risk signature based on m6A regulator expressions could be used to predict prognosis and were associated with the immune microenvironment in gliomas. Additionally, the risk stratification could facilitate the prediction of the efficacy of chemoradiotherapy and might be associated with the efficacy of immunotherapy. These findings indicated that m6A regulators might be potential biomarkers indicating the prognosis and therapeutic efficacy for patients with gliomas and were associated with glioma immune microenvironment. Further studies are needed to explore regulatory mechanisms of m6A regulators in glioma progression and therapeutic efficacy.

## Data Availability Statement

The original contributions presented in the study are included in the article/Supplementary Material, further inquiries can be directed to the corresponding authors.

## Author Contributions

ZL and CL conceived, designed, and supervised the study. SX and LT drafted the manuscript. SX, LT, and GD collected the data. SX performed all data analysis. All authors reviewed and approved the final manuscript.

## Conflict of Interest

The authors declare that the research was conducted in the absence of any commercial or financial relationships that could be construed as a potential conflict of interest.

## Footnotes

1 http://www.cgga.org.cn/

2 https://portal.gdc.cancer.gov/

3 https://cistrome.shinyapps.io/timer/

## Supplementary Material

Supplementary Figure 1 Multivariate Cox analysis of six candidate genes in glioma patients. ^∗^*p* < 0.05; ^∗∗^*p* < 0.01; ^∗∗∗^*p* < 0.001.

Supplementary Figure 2 Survival analysis of patients in age and gender subgroups in four datasets. **(A–D)** Kaplan–Meier analysis of patients with high risk score and low risk score in age and gender subgroups in CGGA325 **(A)**, CGGA693 **(B)**, TCGA **(C)**, and Rembrandt **(D)** datasets.

Supplementary Figure 3 Effect of copy number alternations of m6A regulators on immune cell infiltrations. **(A–D)** Effect of copy number alternations of *ALKBH5* **(A)**, *IGF2BP3* **(B)**, *KIAA1429* **(C)**, and *YTHDF2* **(D)** on immune cell infiltrations. ^∗^*p* < 0.05; ^∗∗^*p* < 0.01; ^∗∗∗^*p* < 0.001.

## Abbreviations

m6A: N6-methyladenosine methylation
CGGA: Chinese Glioma Genome Atlas
TCGA: The Cancer Genome Atlas
ESTIMATE: Estimation of Stromal and Immune cells in Malignant Tumor tissues using Expression data
TIMER: Tumor Immune Estimation Resource
CNAs: copy number alternations
IDH: isocitrate dehydrogenase
WHO: World Health Organization
MGMT: O-6-methylguanine-DNA methyltransferase
METTL: methyltransferase like
RBM: RNA-binding motif
YTHDC: YTH domain-containing
YTHDF: YTH domain family
IGF2BP: insulin-like growth factor-2 mRNA-binding proteins
FTO: fat mass and obesity-associated protein
ALKBH5: alkB homolog 5
LASSO: least absolute shrinkage and selection operator
Vif: variance inflation factor
ROC: receiver operating characteristic
AUC: area under curve.
