# Supplementary figures and images for: Expression of m6A Regulators Correlated With Immune Microenvironment Predicts Therapeutic Efficacy and Prognosis in Gliomas
Source: Front Cell Dev Biol. 2020 Nov 10;8:594112. doi: 10.3389/fcell.2020.594112 (PMC7683617; doi:10.3389/fcell.2020.594112)

# Hazard ratio

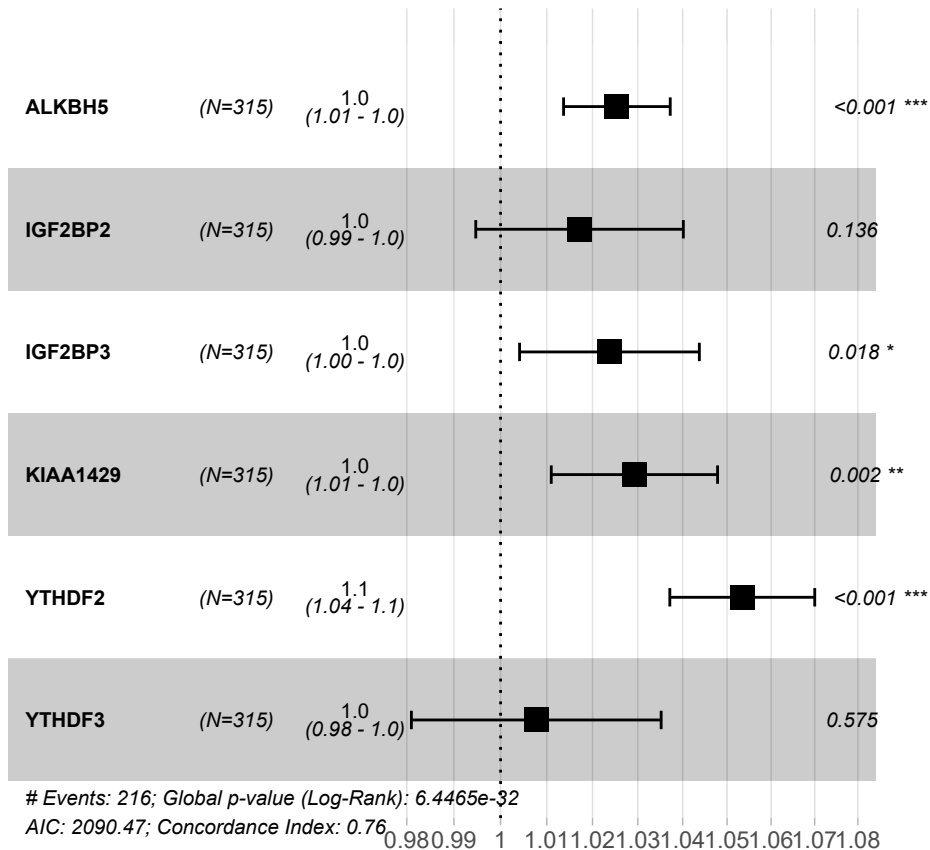

Supplement: Supplementary Figure 1 — Multivariate Cox analysis of six candidate genes in glioma patients. ∗p < 0.05; ∗∗p < 0.01; ∗∗∗p < 0.001. [file Image_1.PDF]

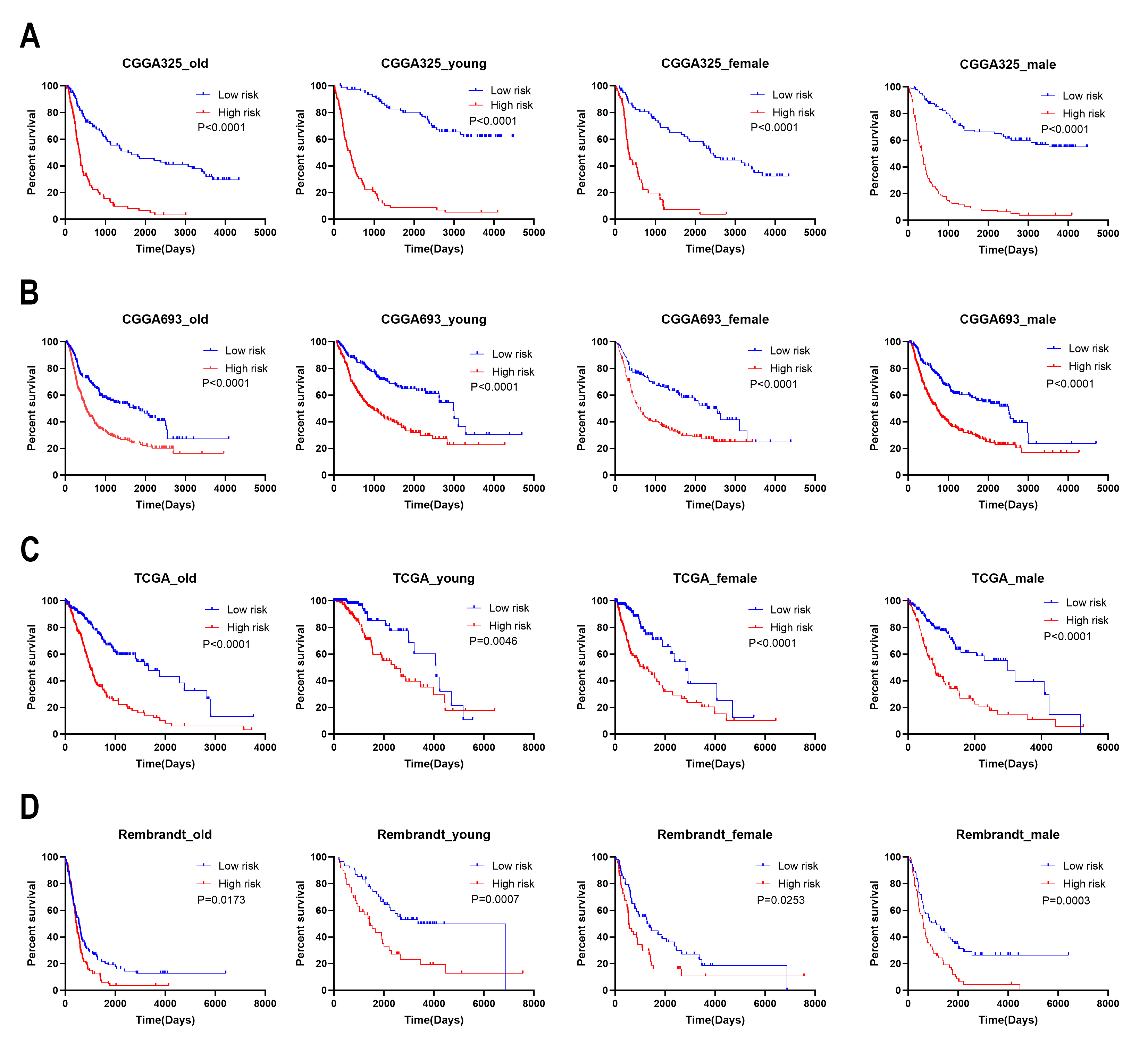

Supplement: Supplementary Figure 2 — Survival analysis of patients in age and gender subgroups in four datasets. (A–D) Kaplan–Meier analysis of patients with high risk score and low risk score in age and gender subgroups in CGGA325 (A), CGGA693 (B), TCGA (C), and Rembrandt (D) datasets. [file Image_2.TIF]

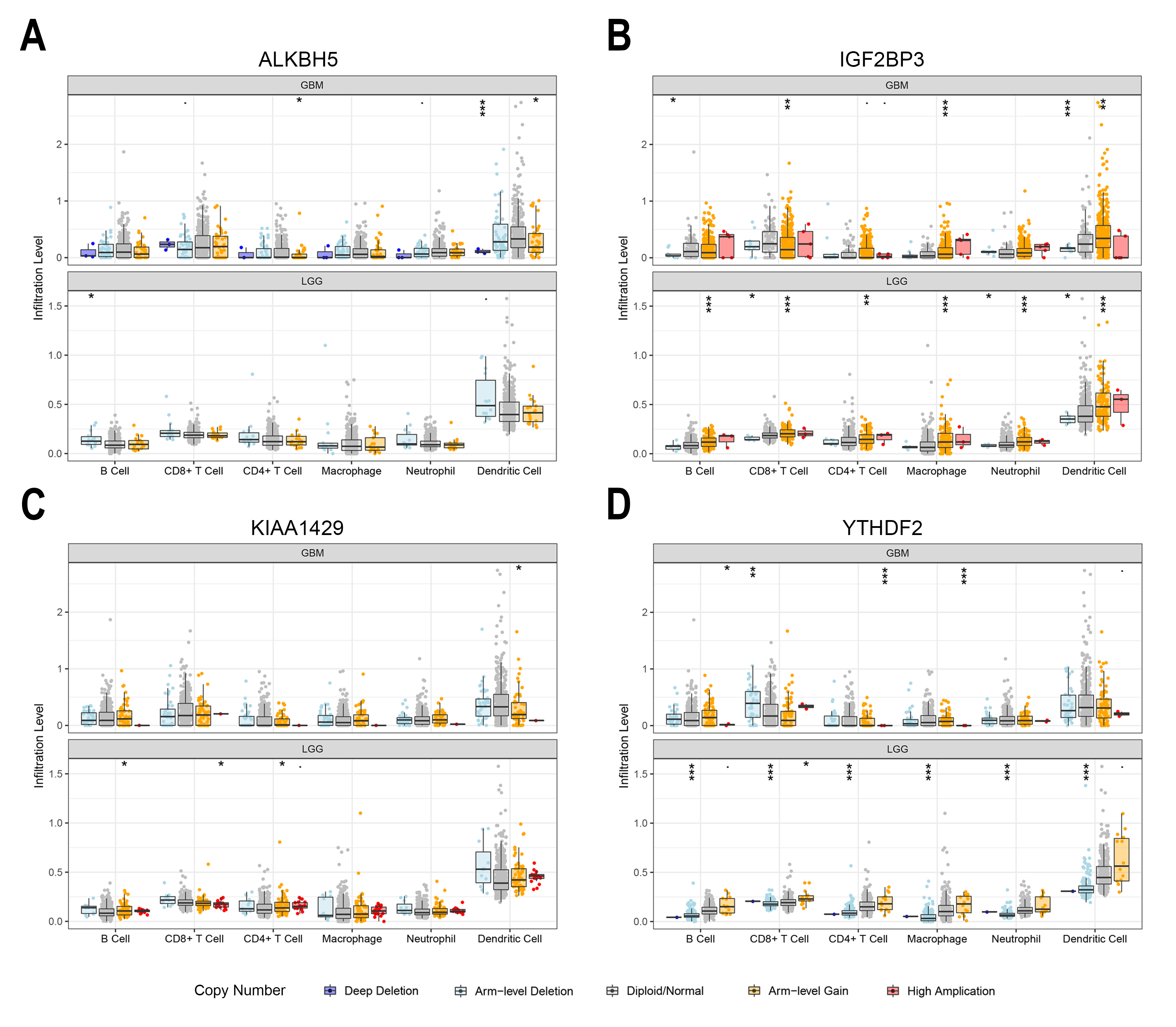

Supplement: Supplementary Figure 3 — Effect of copy number alternations of m6A regulators on immune cell infiltrations. (A–D) Effect of copy number alternations of ALKBH5 (A), IGF2BP3 (B), KIAA1429 (C), and YTHDF2 (D) on immune cell infiltrations. ∗p < 0.05; ∗∗p < 0.01; ∗∗∗p < 0.001. [file Image_3.TIF]
